# The DMRTA1-SOX2 positive feedback loop promotes progression and chemotherapy resistance of esophageal squamous cell carcinoma

**DOI:** 10.32604/or.2023.030184

**Published:** 2023-09-15

**Authors:** RUI ZHANG, PENG ZHOU, XIA OU, PEIZHU ZHAO, XIJING GUO, MIAN XI, CHEN QING

**Affiliations:** 1 School of Pharmaceutical Sciences and Yunnan Key Laboratory of Pharmacology for Natural Products, Kunming Medical University, Kunming, 650500, China; 2 Department of Radiotherapy, The First Affiliated Hospital of Kunming Medical University, Kunming, 650032, China; 3 Department of Pharmacy, The Second Affiliated Hospital of Kunming Medical University, Kunming, 650101, China; 4 Medical School, Kunming University of Science and Technology, Kunming, 650504, China; 5 Department of Radiation Oncology, Sun Yat-sen University Cancer Center, State Key Laboratory of Oncology in South China, Collaborative Innovation Center for Cancer Medicine, Guangzhou, 510060, China

**Keywords:** Esophageal squamous cell carcinoma, DMRTA1, SOX2, Chemotherapy resistance, Immune escape

## Abstract

Esophageal squamous cell carcinoma (ESCC) is among the most prevalent causes of cancer-related death in patients worldwide. Resistance to immunotherapy and chemotherapy results in worse survival outcomes in ESCC. It is urgent to explore the underlying molecular mechanism of immune evasion and chemoresistance in ESCC. Here, we conducted RNA-sequencing analysis in ten ESCC tissues from cisplatin-based neoadjuvant chemotherapy patients. We found that DMRTA1 was extremely upregulated in the non-pathologic complete response (non-pCR) group. The proliferation rate of esophageal squamous carcinoma cells was markedly decreased after knockdown of DMRTA1 expression, which could increase cisplatin sensitivity in ESCC. Additionally, suppression of DMRTA1 could decrease the immune escape of esophageal squamous carcinoma cells. Further mechanistic studies suggest that DMRTA1 can promote its expression by binding to the promoter of SOX2, which plays important roles in the progression and chemoresistance of ESCC in the form of positive feedback. Therefore, DMRTA1 could be a potential target to suppress immune escape and overcome chemoresistance in ESCC.

## Introduction

Esophageal carcinoma is common worldwide, with more than 480,000 new cases diagnosed yearly [1]. Esophageal carcinoma is mainly composed of two histopathological types, esophageal adenocarcinoma and esophageal squamous cell carcinoma (ESCC) [2]. At present, surgery combined with perioperative chemoradiotherapy is the main treatment for advanced ESCC, and its efficacy is gradually being recognized [3]. However, the chemotherapy responses of ESCC patients vary from person to person [4].

The development of drug resistance limits the effectiveness of chemotherapy in patients with ESCC. Therefore, exploring the underlying molecular mechanism of chemoresistance in ESCC is urgent.

Chemotherapy resistance in ESCC involves many mechanisms. Genetic amplification of the follistatin-like gene FSTL1 frequently occurs in ESCC, which results in the overexpression of FSTL1. FSTL1 drives cell proliferation, clonogenicity, migration, invasion, self-renewal, *in vitro* cisplatin resistance, tumorigenicity, and distant metastasis in ESCC [5]. Binding of UBQLN4 to ubiquitinated MRE11A increases the degradation of MRE11A, thereby regulating MRE11A protein expression when DNA is damaged, which promotes the cisplatin resistance of ESCC [6]. IncRNA LINC00261 increases sensitivity to cisplatin treatment via the miR-545-3p/MT1M axis in ESCC [7]. Upregulated HOXB7 expression is related to poor chemotherapy response and cisplatin resistance and can be a biomarker in predicting chemotherapy sensitivity in ESCC [8]. Additionally, the tumor microenvironment (TME) is involved in chemotherapy resistance and the occurrence of ESCC [9]. Nevertheless, the landscape of the molecular mechanisms that trigger resistance to chemotherapy in ESCC is unclear.

Here, we conducted RNA-sequencing analysis in ten ESCC tissues from patients receiving cisplatin-based neoadjuvant chemotherapy. We found that DMRT-like family A1 (DMRTA1) was extremely upregulated in the non-pathologic complete response (non-pCR) group, which was further validated by immunohistochemical staining, qPCR, and western blot analysis. The ability of DMRTA1 to promote tumor progression and drug resistance in ESCC was tested by multiple experiments. The proliferation rate of ESCC cells was markedly decreased and increased after inhibition and overexpression of DMRTA1, respectively. Knockdown of DMRTA1 could promote the remarkable expansion of sensitivity to cisplatin. Mechanistically, our data revealed that the DMRTA1 and SOX2 positive feedback loop promotes tumor progression as well as chemotherapy resistance in ESCC. Overall, this study discovered the function and mechanism of DMRTA1 in ESCC progression and chemoresistance.

## Materials and Methods

### Clinical specimens

ESCC samples and their adjacent nonmalignant controls were acquired from The First Affiliated Hospital of Kunming Medical University and then used for HE staining. All the samples were tested and confirmed by two professional pathologists. The entire study was approved by the Ethics Committee of the above hospital, and the Declaration of Helsinki was strictly observed. Informed consent was obtained from all ESCC patients before the project.

### Cell culture

ATCC library-derived Eca-109, TE-1, EC9706, KYSE-450, TE-13 and HEEC cells were used and cultured according to the instructions from ATCC. All cells were verified by DNA fingerprinting, and mycoplasma infection was tested before the experiments.

### qRT‒PCR analysis

TRIzol reagent (Invitrogen) was adopted for RNA extraction. The SYBR Premix Ex Taq Kit was adopted in this detection (TaKaRa, Japan). DMRTA1 primer sequence, F: 5′-GCAGAGACCGAGGCGTTAG-3′, R: 5′-AACCTGCATCCCCGATGGTA-3′. The primers for SOX2 are F: 5′-GCCGAGTGGAAACTTTTGTCG-3′, R: 5′-GGCAGCGTGTACTTATCCTTCT-3′. The primers for GAPDH are F: 5′-CTGGGCTACACTGAGCACC-3′, R: 5′-AAGTGGTCGTTGAGGGCAATG-3′.

### RNA-seq

The Illumina HiSeq 2500 platform was adopted for RNA-seq. Total RNA was obtained and quantified using a NanoDrop spectrophotometer and Agilent Bioanalyzer. Poly(A) mRNA was enriched, fragmented, and transcribed into cDNA. Then, cDNA libraries were prepared and sequenced. Trimmomatic and TopHat2 software was adopted in the analysis and alignment of raw sequencing data.

### Western blot analysis

RIPA and PMSF were used for protein extraction. Then, the isolated proteins from each group were applied to the PVDF membrane at 300 mA for 90 min. We incubated the sections with anti-DMRTA1 (1:10^3^, Novus), anti-SOX2 (1:10^3^, CST) and anti-GAPDH (1:10^3^, CST) primary antibodies at 4°C and then with secondary antibodies for one hour at room temperature.

### Cell counting kit-8 (CCK-8) assay

Eca-109 and KYSE-450 ESCC cells (3 × 10^3^) were resuspended, seeded and cultivated in 96-well plates for 3 days in an incubator, and 10 μl of CCK-8 reagent was added to the plate and replaced in the incubator. Two hours later, the absorbance at 450 nm was tested.

### Transwell assay

Eca-109 ESCC cells (2 × 10^4^) were resuspended and replaced in the upper chambers (no FBS) and medium (20% FBS) in the lower part. Then, cells in the upper part were removed. Migrated cells were stained and photographed under a microscope.

### Wound-healing assay

The wound-healing assay is a commonly used method to investigate cell migration and wound closure. Briefly, cells were seeded in a suitable medium until they reached confluence. A wound was created in the cells. Images of the wound were taken at the beginning of the experiment and at regular intervals during the healing process. The distance between the wound was detected through image analysis software to quantify the rate of wound closure.

### Promoter and luciferase activity assay

We cloned the promoter sequences into the pGL3-basic vector. Then, the DMRTA1 and SOX2 promoter regions (−2000 to TSS) were amplified. The predicted binding sites were mutated. Established reporting plasmids and DMRTA1 or SOX2 overexpression plasmids were cotransfected into ESCC cells. Forty-eight hours later, 5000 Eca-109 and KYSE-450 ESCC cells were added to 96-well plates. Then, the relative activity of the luciferase enzyme was tested.

### Subcutaneous xenograft model

For the subcutaneous xenograft model, 5 × 10^6^ cells were subcutaneously injected into nude mice (200 μL, BALB/c, 5 weeks, Beijing Vital River Laboratories, China). When tumors were palpable, the mice were treated with cisplatin. Every 8 days, tumor size was measured. The calculation formula for tumor volume (V) was 0.5 × length × width^2^. Twenty-four days later, the test was ended. Tumor samples were collected for weighing for all groups. Then, through the tail vein, 10^5^ cells were injected to construct a lung metastasis model. Eight weeks later, the test was ended to obtain the lung samples. Lung metastatic lesions were quantified by HE staining.

### Statistical analysis

SPSS 24.0 software was chosen for data analysis. All data are shown as the mean ± standard deviation. Student’s *t* test was used to analyze data between different groups; when *p* < 0.05, the difference was statistically significant.

## Results

### DMRTA1 is upregulated in chemotherapy-resistant ESCC

**Figure 1:**
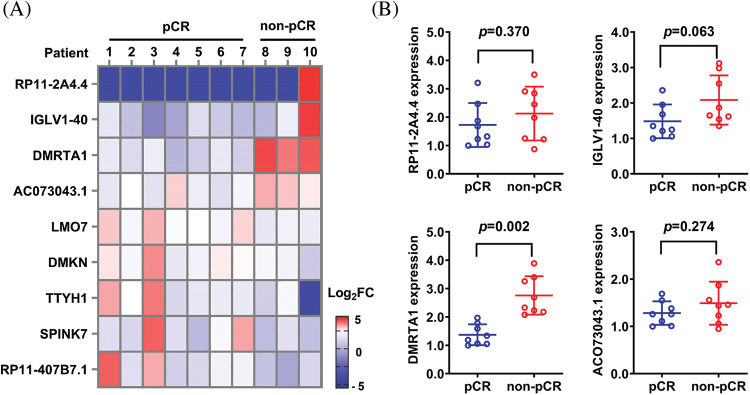
RNA sequencing screening identified DMRTA1 as a resistance gene in ESCC. (A) The heatmap shows the RNA expression profile of seven pCR patients and three non-pCR patients. Red indicates high expression, and blue indicates the opposite. (B) The relative expression of the top four upregulated genes was validated in 16 ESCC samples with a pCR or non-pCR outcome after neoadjuvant chemotherapy.

**Figure 2:**
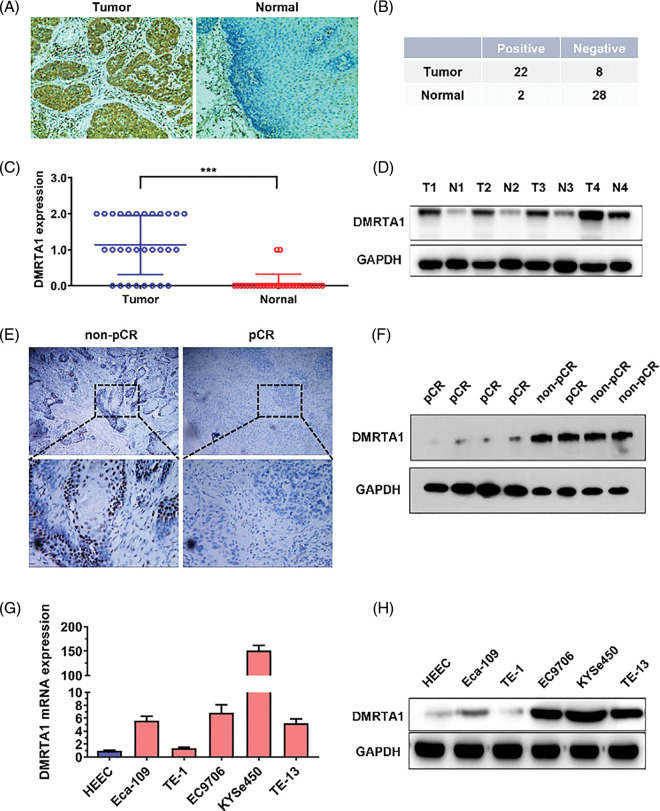
DMRTA1 is overexpressed in chemotherapy-resistant ESCC. (A) Representative immunohistochemical staining images of DMRTA1 in ESCC and control esophageal epithelial samples. (B, C) The positivity and expression of DMRTA1 in ESCC and control esophageal epithelial samples. (D) DMRTA1 expression in ESCC and paired normal adjacent esophageal epithelial tissues, evaluated by western blot analysis. (E) Representative immunohistochemical staining images of DMRTA1 expression in pCR (sensitive) and non-pCR (resistant) ESCC tissues. (F) The expression level of DMRTA1 in pCR (sensitive) and non-pCR (resistant) ESCC tissues, assessed by western blot analysis. (G) DMRTA1 was highly expressed in esophageal cancer cells, as validated by qPCR analysis. (H) DMRTA1 was highly expressed, as validated by western blot analysis. ***p < 0.001.

To study the chemotherapy resistance gene expression profile of ESCC, we conducted RNA sequencing analysis in ten ESCC tissues from cisplatin-based neoadjuvant chemotherapy patients (Fig. 1A). Among them, seven patients achieved pathological complete response (pCR), while three patients did not achieve pCR after neoadjuvant chemotherapy. We then used another 16 ESCC samples for further validation and found that DMRTA1 was highly upregulated in the non-pCR group (Fig. 1B). As detected by immunohistochemical staining, DMRTA1 was overexpressed in ESCC tissues compared with control tissues (Fig. 2A). The positive rate of DMRTA1 was also higher in ESCC tissues (Figs. 2B and 2C). We also found that DMRTA1 was upregulated in ESCC tissues (Fig. 2D and Suppl. S1A). Additionally, we revealed that DMRTA1 had higher expression in the non-pCR group than in the pCR group (Figs. 2E, 2F and Suppl. Fig. S1B). Next, we detected the expression of DMRTA1 in a variety of esophageal cancer cells. Increased DMRTA1 expression was observed in esophageal cancer cells compared to control HEEC cells, as validated by qPCR and western blot analysis (Figs. 2G, 2H and Suppl. Fig. S1C).

### Inhibition of DMRTA1 attenuates proliferation and enhances chemotherapy sensitivity in ESCC *cells*

**Figure 3:**
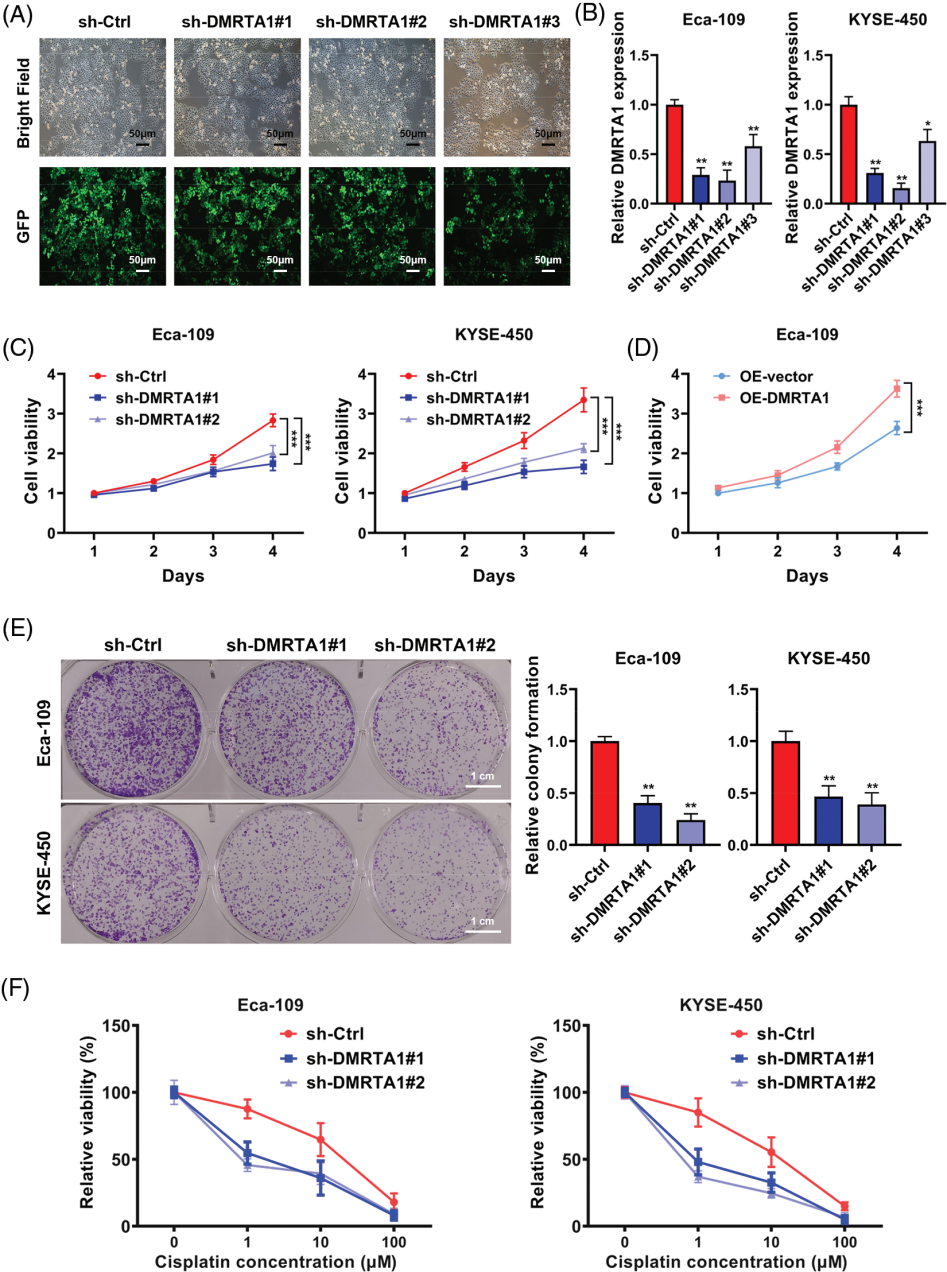
Inhibition of DMRTA1 attenuates proliferation and enhances chemotherapy sensitivity in ESCC cells. (A, B) The efficiency of DMRTA1 shRNA was tested and evaluated via fluorescence microscopy imaging and qRT‒PCR analysis. (C, D) A CCK-8 assay was conducted to assess cell growth. (E) Colony formation experiments were performed. (F) Inhibition of DMRTA1 markedly increased cisplatin sensitivity. *p < 0.05; **p < 0.01; ***p < 0.001.

Functional assays were adopted to investigate DMRTA1 function in ESCC. We constructed stable DMRTA1 suppression and overexpression KYSE-450 and Eca-109 esophageal squamous carcinoma cells via lentivirus infection (Fig. 3A). The efficiency was examined by qPCR (Fig. 3B). The proliferation rate was markedly decreased and increased after inhibition and overexpression of DMRTA1, respectively, as revealed by CCK-8 assays (Figs. 3C and 3D). The colony-generative potential of ESCC cells was suppressed by DMRTA1 inhibition (Fig. 3E). In addition, knockdown of DMRTA1 improved the cisplatin sensitivity of Eca-109 and KYSE-450 esophageal squamous carcinoma cells (Fig. 3F).

### DMRTA1 promotes migration and stemness in ESCC cells

**Figure 4:**
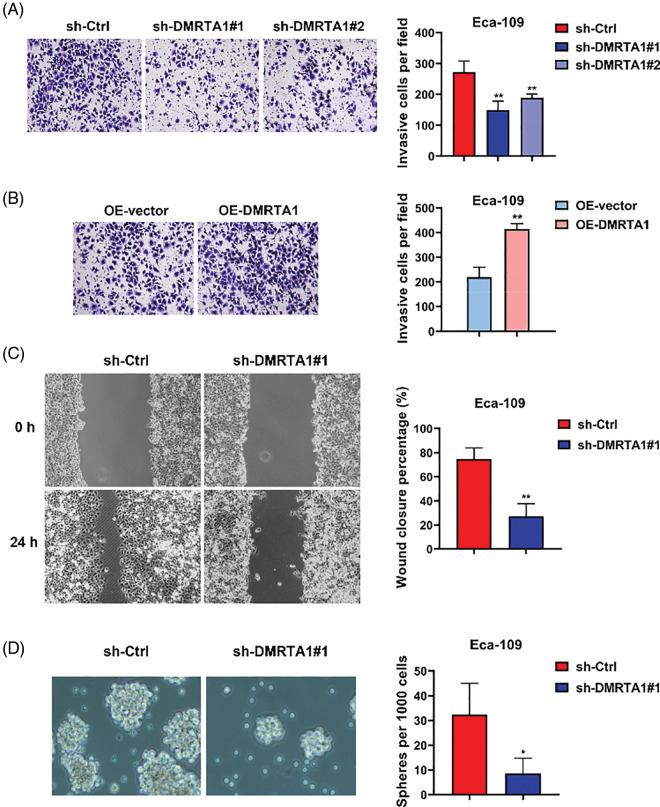
DMRTA1 promotes migration and stemness in ESCC cells. (A) Transwell assays were conducted to assess the metastatic ability of Eca-109 cells after knockdown of DMRTA1. (B) Transwell assays were adopted to assess the metastatic ability of Eca-109 cells after overexpressing DMRTA1. (C) A wound-healing experiment was carried out to assess the migration of cells after inhibition of DMRTA1 in the Eca-109 esophageal squamous carcinoma cell line. (D) Knockdown of DMRTA1 also showed a strong effect on the suppression of ESCC stem cells. *p < 0.05; **p < 0.01.

To clarify the function of DMRTA1 in the metastasis of esophageal squamous carcinoma cells, we performed transwell and wound-healing assays. The migration declined when DMRTA1 was inhibited (Fig. 4A), while the ability increased when DMRTA1 was overexpressed (Fig. 4B). As revealed by the wound-healing experiment, suppression of DMRTA1 attenuated the wound closure percentage in Eca-109 cells (Fig. 4C). Moreover, knockdown of DMRTA1 showed a strong effect on the suppression of cancer stem cells, which led to metastasis and drug resistance of cancer cells (Fig. 4D).

### DMRTA1 confers chemotherapy resistance and metastasis to ESCC in mouse xenograft models

**Figure 5:**
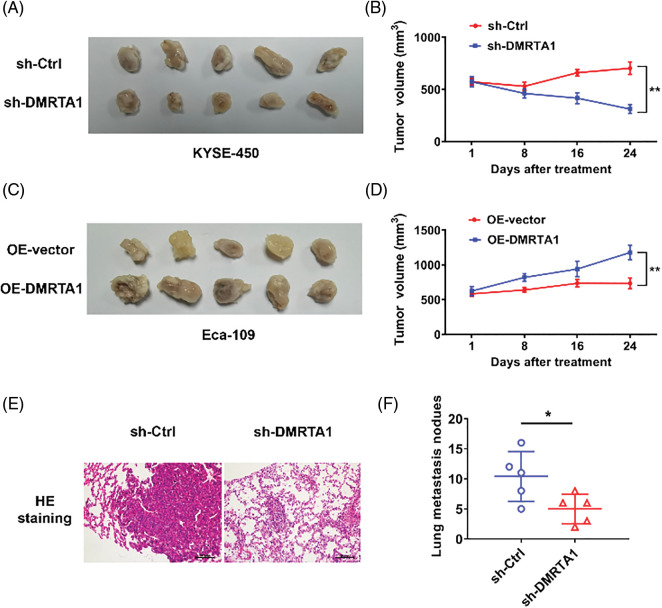
DMRTA1 confers chemotherapy resistance and metastasis to ESCC in mouse xenograft models. (A, B) Cisplatin resistance of ESCC was evaluated after knockdown of DMRTA1. The Eca-109 cell line was used to establish a mouse xenograft model. (C, D) Cisplatin resistance was evaluated after overexpressing DMRTA1 in ESCC. The KYSE-450 cell line was used to establish a mouse xenograft model. (E) Representative HE-stained sections of lung metastases. (F) The number of lung metastases was counted and recorded. *p < 0.05; **p < 0.01.

Mouse xenograft models were then established to validate the influence of DMRTA1 on the chemotherapy resistance and metastasis of ESCC. DMRTA1 silencing decreased the tumor growth rate in Eca-109 esophageal squamous carcinoma cells after treatment with cisplatin (Figs. 5A and 5B). Overexpression of DMRTA1 also increased tumor growth and volume in cisplatin-treated nude mice (Figs. 5C and 5D). In addition, DMRTA1 silencing restrained the metastatic ability of Eca-109 cells in lung metastasis experiments *in vivo*, indicating that DMRTA1 plays a role in ESCC metastasis (Figs. 5E and 5F).

### The DMRTA1 and SOX2 positive feedback loop promotes progression and chemotherapy resistance of ESCC

**Figure 6:**
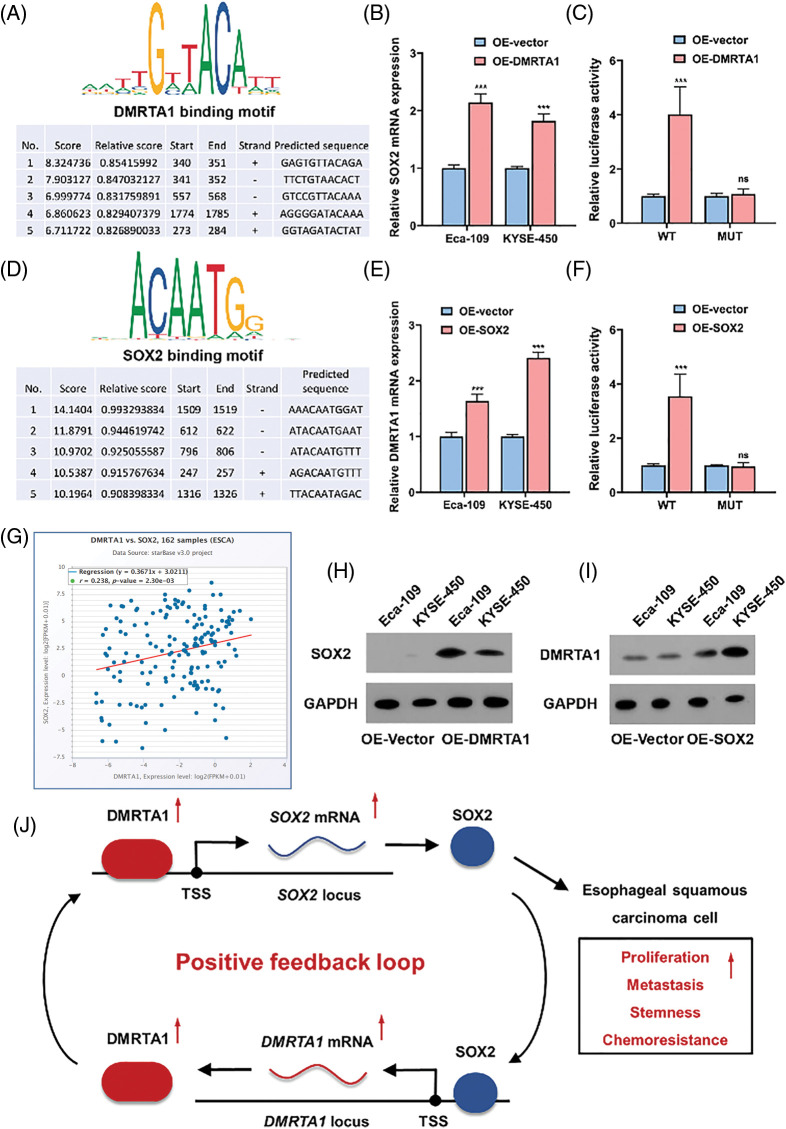
The DMRTA1 and SOX2 positive feedback loop promotes progression and chemotherapy resistance in ESCC. (A) The JASPAR algorithm was adopted for sequence and binding motif prediction. Several binding sites of DMRTA1 on the promoter DNA sequence of the SOX2 locus are shown. (B) Overexpression of DMRTA1 increased the SOX2 mRNA levels in both Eca-109 and KYSE-450 esophageal squamous carcinoma cell lines. (C) The fluorescence value was increased when DMRTA1 was overexpressed in the wild-type reporter group; however, it had no effect in the mutated reporter group. (D) The prediction of SOX2 sequences and binding motifs. Several binding sites of SOX2 on the promoter DNA sequence of the DMRTA1 locus are shown. (E) Overexpression of SOX2 increased the DMRTA1 mRNA levels in both Eca-109 and KYSE-450 esophageal squamous carcinoma cell lines. (F) The fluorescence value was increased when SOX2 was overexpressed in the wild-type reporter group; however, SOX2 overexpression had no effect in the mutated reporter group. (G) The correlation between DMRTA1 and SOX2 expression in ESCC, according to TCGA database. Spearman test was used. (H) Overexpressed DMRTA1 increased SOX2 protein expression in Eca-109 and KYSE-450 cells. (I) Overexpressed SOX2 increased DMRTA1 protein expression in Eca-109 and KYSE-450 esophageal squamous carcinoma cells. (J) The schematic diagram illustrates the biological mechanism of the DMRTA1 and SOX2 positive feedback loop in promoting the progression and chemotherapy resistance of ESCC. ***p < 0.001.

As a transcription factor, DMRTA1 regulates biological processes via transcriptional activation of key downstream genes in cells. To elucidate the potential mechanism involved in DMRTA1 in ESCC progression as well as cisplatin resistance, we used the JASPAR algorithm to predict the downstream pathway (Fig. 6A). We found that there are several binding sites of DMRTA1 on the promoter DNA sequence of the SOX2 locus (Fig. 6A). This molecule is an important regulator of cell fate during cancer development and cell reprogramming and is important in the renewal of cancer stem cells and drug resistance [10–12]. Overexpression of DMRTA1 increased SOX2 mRNA expression in both Eca-109 and KYSE-450 esophageal squamous carcinoma cells (Fig. 6B). Then, we assessed the binding sites of DMRTA1 on the SOX2 promoter. The results showed that the fluorescence value was increased when DMRTA1 was overexpressed in the wild-type reporter group; however, it had no effect in the mutated reporter group (Fig. 6C). Interestingly, we found that SOX2 could bind to the sequence of the DMRTA1 promoter by using the JASPAR predictive algorithm (Fig. 6D). Exogenous overexpression of SOX2 elevated the DMRTA1 mRNA levels in both Eca-109 and KYSE-450 esophageal squamous carcinoma cell lines (Fig. 6E). As revealed by luciferase reporting assays, the relative value of luciferase activity was increased after transfection with the SOX2 overexpression plasmid in the wild-type reporter group; however, it had no effect in the mutated reporter group (Fig. 6F). Additionally, there was a strong positive correlation between DMRTA1 and SOX2 expression in ESCC, according to TCGA database (Fig. 6G). Overexpression of DMRTA1 increased the expression level of SOX2 protein (Figs. 6H and Suppl. Fig. S1D). Consistently, the DMRTA1 protein expression level was also elevated after overexpressing SOX2, as shown by western blot assays (Figs. 6I and Suppl. Fig. S1E). Generally, our results revealed that the DMRTA1 and SOX2 positive feedback loop promotes the progression and chemotherapy resistance of ESCC (Fig. 6J).

## Discussion

Drug resistance is an insurmountable obstacle in curing cancer patients [13]. Thus, elucidating the simultaneous resistance mechanisms of drugs that have different targets or chemical structures has been very important in recent decades. There are several resistance mechanisms involved in cancer treatment. For example, one type of self-renewing cancer cell, cancer stem cells, shows strong resistance to chemotherapy and avoids being killed by drugs in a variety of cancers [14,15]. The stable epigenetic state of tumor cells could drive several steps of epigenetic fixation in gene expression, which has a vital role in clinical strategies [16]. Resistant cells maintain high activity of scavenging or antioxidant enzymes, which help them eliminate reactive oxygen species (ROS) in cells [17,18]. Increasing evidence has demonstrated that the internal relationship between the tumor microenvironment (TME) and tumor cells exerts a strong influence on the drug resistance of cancer [19–24]. Elucidating the mechanism of chemotherapy resistance is an important strategy to solve drug resistance.

Here, RNA-sequencing analysis of seven pCR and three non-pCR ESCC tissues from cisplatin-based neoadjuvant chemotherapy-treated ESCC patients was performed. We first discovered that DMRTA1 was upregulated in the non-pCR group. Knockdown of DMRTA1 distinctly increased the sensitivity of ESCC cells to cisplatin. Mechanistically, our data revealed that the DMRTA1 and SOX2 positive feedback loop promotes chemotherapy resistance and progression in ESCC.

As a transcription factor, the DMRTA1 protein contains a DNA sequence-specific double-stranded binding domain that enables DMRTA1 to activate the transcription of downstream genes [25]. Dmrta1 was reported to regulate testis spermatogenesis and ovarian follicle development in the early years [25,26]. Studies have revealed that DMRTA1 plays vital roles in various diseases. Dmrta1 promotes the development of the mammalian telencephalon by regulating proneural gene expression downstream of Pax6 [27]. Single nucleotide polymorphism of DMRTA1 was found to be closely related to type 2 diabetes [28]. DMRTA1 was associated with poorer prognosis and was characterized by significant elevation of M2 subtype macrophage infiltration and immune escape biomarkers and higher cytolysis and immune evasion scores in isocitrate dehydrogenase wild-type diffuse lower-grade glioma [29]. Through a comprehensive analysis of urothelial cancer cell lines, DMRTA1 was found to be associated with platinum resistance by activating the DNA repair pathway [30]. In our study, DMRTA1 drives chemotherapy resistance via transcriptionally activating SOX2 expression in ESCC. SOX2 is an important regulator of cell fate during tumor development and cell reprogramming, which is closely related to stem cell renewal and drug resistance [31–33]. Regulated by a histone deacetylase 2-mediated epigenetic mechanism, the METTL14-miR-99a-5p-tribble 2 positive feedback loop promotes radiotherapy resistance and cancer stem cell persistence in ESCC [34]. Interestingly, we also found that DMRTA1 and SOX2 could enhance the expression of each other, which forms a positive feedback regulation circuit. There are many positive feedback regulatory pathways in the molecular mechanisms of tumor progression [35]. The KLF5-mediated positive feedback loop promotes immune evasion in metastatic breast cancer [36]. HLA-E:CD94-NKG2A engages the immune checkpoint RGS18 to protect CTCs against NK-mediated immune surveillance [37].

In conclusion, this study elucidated the function and mechanism of DMRTA1 in the progression and chemoresistance of ESCC. DMRTA1 could be a potential ESCC therapeutic target to overcome chemoresistance in the future.

## Supplemental Materials

**Figure S1:**
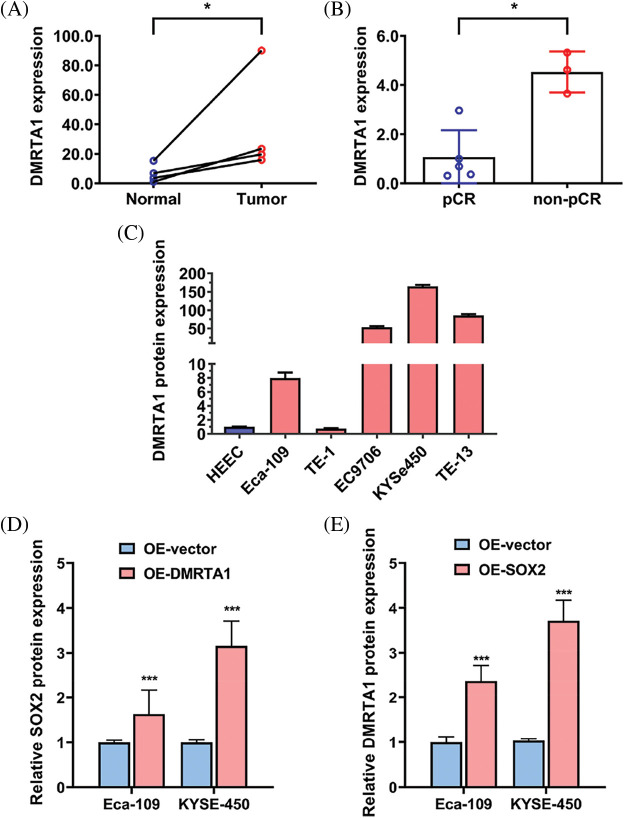
(A) DMRTA1 expression in ESCC and paired normal adjacent esophageal epithelial tissues, evaluated by western blot analysis. (B) The expression level of DMRTA1 in pCR (sensitive) and non-pCR (resistant) ESCC tissues, assessed by western blot analysis. (C) DMRTA1 was highly expressed in esophageal cancer cells, as validated by western blot analysis. (D) Overexpressed DMRTA1 increased SOX2 protein expression in Eca-109 and KYSE-450 cells. (E) Overexpressed SOX2 increased DMRTA1 protein expression in Eca-109 and KYSE-450 esophageal squamous carcinoma cells. *p < 0.05; ***p < 0.001.

## Data Availability

Not applicable.
